# Supplementary material for: Imaging-based intelligent spectrometer on a plasmonic rainbow chip
Source: Nat Commun. 2023 Apr 5;14:1902. doi: 10.1038/s41467-023-37628-0 (PMC10076426; doi:10.1038/s41467-023-37628-0)
Supplement: Supplementary file 1 — Supplementary Information [file 41467_2023_37628_MOESM1_ESM.pdf]

Supplementary Information for
Imaging-based intelligent spectrometer on a plasmonic rainbow chip

Dylan Tua[1*], Ruiying Liu[1*], Wenhong Yang[2*], Lyu Zhou[1], Haomin Song[2], Leslie Ying[†,1], Qiaoqiang Gan[‡,1,2]

[1] *Electrical Engineering, University at Buffalo, The State University of New York, Buffalo, NY 14260*

[2] *Material Science Engineering, Physical Science Engineering Division, King Abdullah University of Science and Technology, Thuwal 23955-6900, Saudi Arabia*

* These authors contributed equally to this work

† Email: leiying@buffalo.edu; ‡ Email: qiaoqiang.gan@kaust.edu.sa

Table of Contents:
S1. Fabrication of the 1D rainbow chip
S2. Numerical modeling of the rainbow trapping structure
S3. Conventional methods for spectrum reconstruction
S4. Deep learning spectrum reconstruction method
S5. Reconstruction of broadband spectra
S6. Spectral resolution of reconstructed spectra
S7. Reconstruction of two-peak spectra
S8. Resolving adjacent two peaks using Rayleigh criterion
S9. Fabrication of the 2D rainbow chip
S10. Modeling of the 2D rainbow chip
S11. Spectral range of a given 2D rainbow chip
S12. Deep learning method for polarized spectra reconstruction
S13. Training and testing procedure for spectrum reconstruction of the image-based spectrometer
S14. Deep learning method for polarization state prediction
S15. Training and testing procedure for ORD sensing of the image-based spectrometer
S16. Experimental measurement of ORD using conventional methods
S17. Comparative FOMs of the proposed scheme with previous works

List of Figures and Tables:
Figure S1. SEM image of the 1D rainbow chip.
Figure S2. Zoom-in images of the 1D rainbow chip at short and long period regions.
Figure S3. Numerical modeling of the one-dimensional rainbow chip.
Figure S4. Flowchart of the conventional spectrum reconstruction method.
Figure S5. Flowchart of the deep learning spectrum reconstruction method.
Figure S6. NMSE comparison between the deep learning and conventional reconstruction methods.
Figure S7. NMSE for the reconstructed broadband spectra using the proposed deep learning method.
Figure S8. Reconstruction of two adjacent peaks to determine the resolution using the Rayleigh criterion.
Figure S9. Numerical modeling of the 2D chirped grating.
Figure S10. Spectral range determination of the 2D graded grating.
Figure S11. Flowchart of the polarized spectrum reconstruction.
Figure S12. Flowchart of the single wavelength polarization prediction.
Figure S13. Flowchart of the multiple wavelength polarization predictions.
Table S1. Training and testing data parameters for 1D spectrum reconstruction.
Table S2. Parameters of the training data collected for broadband spectrum reconstruction.
Table S3. Parameters of the testing data collected for broadband spectrum reconstruction.
Table S4. Training and testing data parameters for spectral resolution.

Table S5. Training and testing data parameters for two-peak spectrum reconstruction.
Table S6. Parameters of the training and testing data collected for spectrum reconstruction.
Table S7. Training and testing data parameters for single-peak illumination for ORD sensing.
Table S8. Training and testing data parameters for double-peak illumination for ORD sensing.
Table S9. Training and testing data parameters for triple-peak illumination for ORD sensing.
Table S10. Deviation of reconstructed angles at different wavelengths in Fig. 5f-g in the main text.
Table S11. Comparative FOMs of the proposed scheme and schemes from several references.

Note S1: Fabrication of the 1D rainbow chip

Fabrication for the 1D rainbow chip began with deposition of a 300 nm-thick Ag film on a glass slide via electron beam evaporation. Focus ion beam (FIB) milling was used to etch the graded grating patterns into the Ag film. The graded patterns used to fabricate the grating were designed on the NanoPatterning & Visualization Engine (NPVE) software connected to the FIB hardware (Zeiss AURIGA CrossBeam). The period of the gratings varied from 244 nm to 764 nm. To form the gradient, the grooves of the grating were fabricated in groups of six. Gratings within a group had equal periods, with the period increasing by 10 nm along the length of the pattern until the longest period was reached.

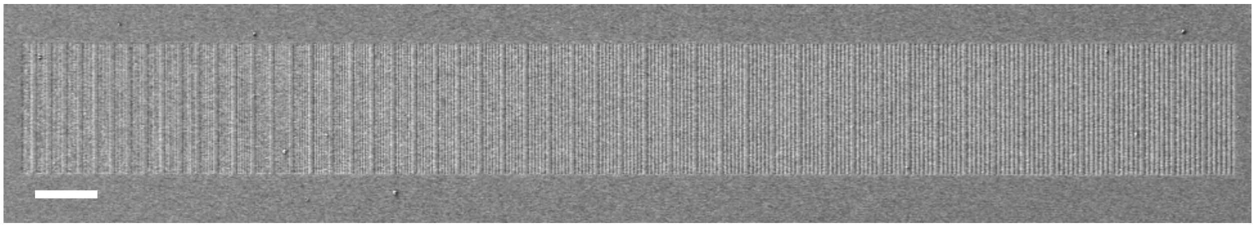

Figure S1. SEM image of the 1D rainbow chip. The scale bar is 10 µm.

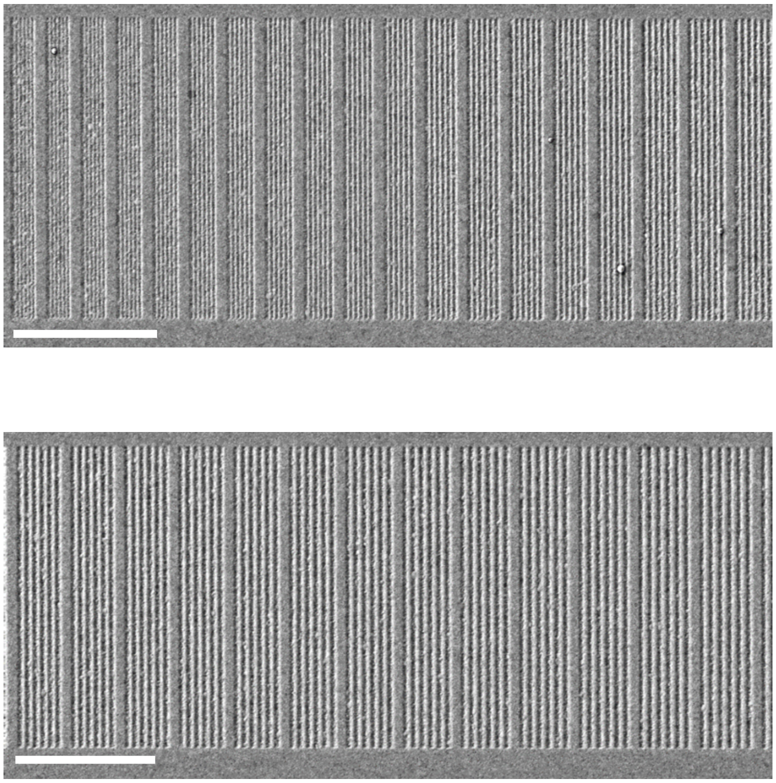

Figure S2. Zoom-in of the 1D rainbow chip at short (top) and long (bottom) period regions. The scale bars are 10 µm.

Note S2: Numerical modeling of the rainbow trapping structure

The simulations of the electric field distribution are calculated by the commercial software Lumerical FDTD Solutions. Perfectly matched layers are applied at boundaries to absorb the outgoing waves. A non-uniform mesh was used with a bulk mesh of 2 nm. As shown in **Fig. S3a**, a transverse magnetic (TM) plane wave is launched to illuminate the graded surface grating which composed from an assembly of multiple 6-groove units with a groove width of 200 nm and depth of 35 nm. The period of the grating changes from 244 nm to 764 nm with increasing of 10 nm along the length of pattern. Under the illumination at the narrow band incident wavelength from 530 nm to 650 nm, the optical intensity distribution in the near-field (see **Fig. S3b-f**) are recorded. One can see that the trapped position in the near field shifts along the graded grating. In particular, a dark reflection pattern can be observed in the far-field, corresponding to the dark bar observed in our microscopic images shown in Fig. 1 of the main text. Therefore, the observed dark bars in the reflection images are closely related to the rainbow trapping effect.

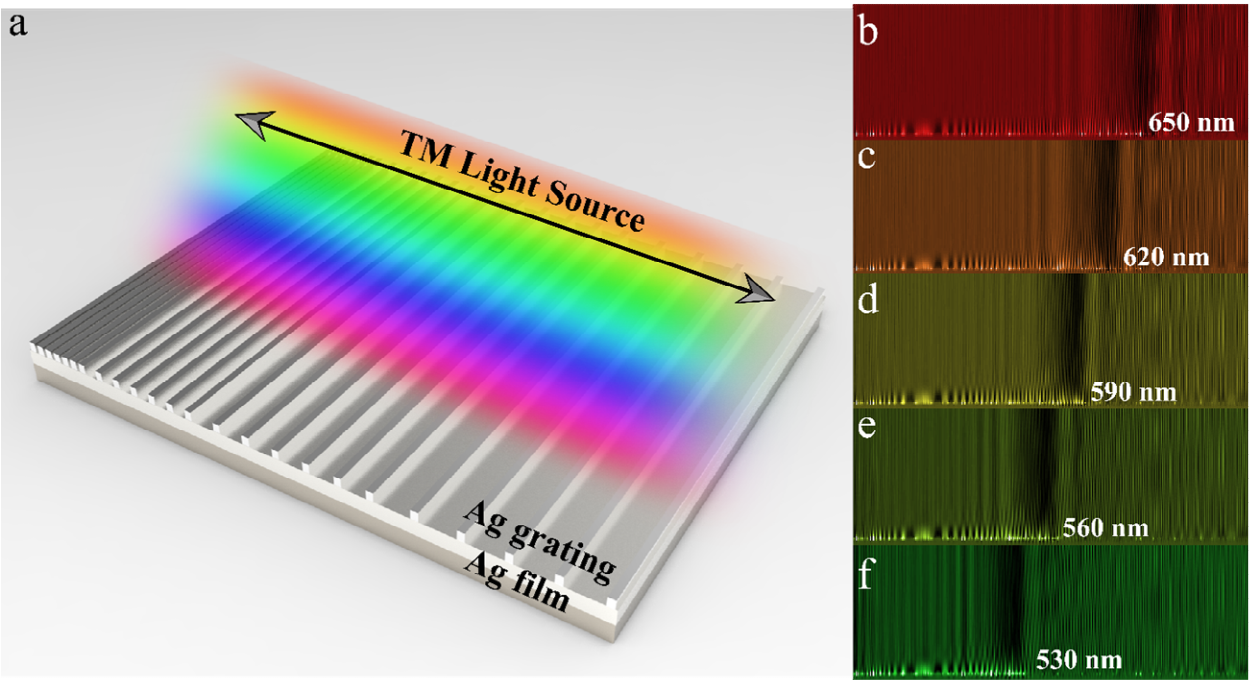

Figure S3. Numerical modeling of the one-dimensional rainbow chip. (a) Schematic of graded surface grating structure illuminated by an incident TM plane wave. (b)-(f) The simulated side-view electric field distributions in near field at incident wavelengths from 530 nm to 650 nm.

Note S3: Conventional methods for spectrum reconstruction

The miniaturization of spectrometer systems usually sacrifices the accuracy of the reconstructed spectra due to the over-simplified optical design and mechanical limit of ultra-compact architectures. Therefore, achieving accurate reconstruction of an unknown spectrum using only images of its spectral resonance is one of the grand challenges in engineering such devices.

In recent years, accurate spectrum reconstruction has become one of the most important procedures required for compact spectrometer systems, as reported in the recent publication of the single-nanowire spectrometer [R1]. The architecture of a conventional system is shown in **Fig. S4**. The resonant spectral pattern (Fig. 1d in the main text) was measured for each of the n photodetector units (Fig. 1b in the main text) as input in **Fig. S4**. The measured pattern for the ith unit is an integral of the incident light spectrum $F(\lambda)$ (Fig. 1e in the main text) multiplied by the corresponding spectral response function $R_i(\lambda)$ for that particular unit (precalibrated) over the spectrometer's operational wavelength range (e.g., from λ_1 to λ_2), which can be represented as a system of linear equations:

$$I_i = \int_{\lambda_1}^{\lambda_2} F(\lambda)R_i(\lambda)d\lambda, \qquad \text{i=1, 2, …n} \tag{1}$$

Spectra reconstruction involves solving the above equations for the unknown spectrum $F(\lambda)$. Before we reconstruct $F(\lambda)$, the spectral response functions $R_i(\lambda)$ of each photodetector is predetermined and must be calibrated. We collect paired incident light spectrum $F(\lambda)$ and resonant spectral patterns I as training data. The spectral response functions R can be determined using L2-regularized optimization problem, as shown in equation (2):

$$\min_{R} \|F * R - I\|_2^2 \tag{2}$$

where I and R are the observed signals, and F is the problem variable. These equations are typically unguaranteed due to the measurement errors in both I_i and R_i [R1-R3], meaning the reconstructed target spectrum can be largely distorted even at a low noise level. Several methods have been used to address the issue of ill-posedness, such as adaptive Tikhonov regularization [R1].

$$\min_{F} \{\|F * R - I\|_2^2 + \lambda\|F\|_2^2\} \tag{3}$$

As shown in equation (3), the algorithm uses a linear combination of Gaussian basis functions with different amplitudes to fit the target spectrum [R1]. However, these methods heavily rely on the accuracy of the estimated spectral response function $R_i(\lambda)$, which is typically unknown. In addition, the regularization, which involves tedious parameter tuning, can introduce bias to the reconstructed spectrum. The computational complexity can be high when solving a large number of equations.

In the main text, we report an AI-based method for the reconstruction of an unknown spectrum to address the above-mentioned challenges. The method does not require knowledge of the spectral response function, thereby avoiding spectral errors caused by the miscalibrated spectral response function $R_i(\lambda)$. The AI-based method can represent any nonlinearity based on the imperfection of the spectrometer, which can estimate the spectrum accurately. Although the training time for the AI-based method is long, the reconstruction time is highly efficient once the model is calibrated.

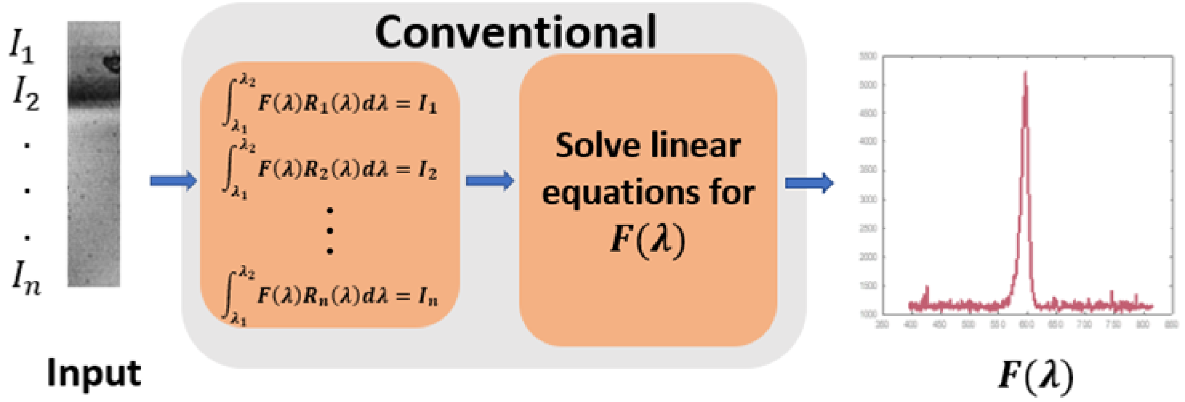

Figure S4. Flowchart of the conventional spectrum reconstruction method.

Note S4: Deep learning spectrum reconstruction method

Deep learning (DL) promises to discover rich, hierarchical models [R4,R5] that represent probability distributions over the kinds of data encountered in artificial intelligence applications. DL is a class of AI-based algorithms that exploit many cascaded layers of nonlinear information processing units to learn the complex relationships among data. By going deeper (i.e., adding layers), the network improves its capability of learning features from higher levels of the hierarchy that were formed by the composition of lower-level features. In this work, we choose deep neural networks due to their ability to learn complex mappings between input and target spectrum, bypassing traditional methods.

The developed deep neural network can find the relationship between the measured resonance pattern I_i and the unknown incident light spectrum $F(\lambda)$, bypassing the traditional linear model in Eq. (1). Although it is well accepted that the relationship is linear according to the optical properties, the measurement errors/noise and ill-posedness of Eq. (1) can make the relationship to be nonlinear. Our deep neural network includes a fully connected multilayer perceptron (MLP) and a convolutional neural network (CNN). **Fig. S5** shows the flowchart of the deep neural network spectral reconstruction method. MLP, one of the simplest forward-structured artificial neural networks, maps a set of input vectors to a set of output vectors. It can be thought of as a directed graph, consisting of multiple fully-connected (FC) layers. Each hidden layer accepts output from the previous layer as input and returns a nonlinear affine transformation as the output. However, a very deep (i.e., many layered) MLP is inefficient, since such high dimensions are prone to redundancies that disregard spatial information. To alleviate the typical overfitting problem for MLP, a CNN was included, which has demonstrated success in processing high-dimensional data (e.g., computer vision). Similar to MLP, the CNN uses an input layer, an output layer, and multiple hidden layers to form a hierarchical structure. However, not all nodes from the previous layer are connected to the nodes in the next layer. The computations performed by the CNN use image patches as the dataflow unit instead of individual pixels, with a linear convolution followed by a nonlinear activation function performed at each node. As such, the inclusion of the CNN significantly improves the performance of the network.

Here we assume that $I \in \mathbb{R}_{\mathrm{a}\times\mathrm{b}}$ is an input image of size $a \times b$ and $F(\lambda) \in \mathbb{R}_{1\times\mathrm{n}}$ is the output light intensity with wavelength λ ranging from λ_1 to λ_n. The relationship between I and $F(\lambda)$ can be expressed as $F(\lambda) = H(I_i; \Theta)$, where H is a nonlinear function represented by the MLP network and Θ is the network parameters. During training (similar to calibration of the spectral response function), multiple incident lights with known, varying spectra are shined on the miniature spectrometer and the corresponding resonant patterns are recorded. Such input and output pairs of the training dataset are used to estimate the network parameters Θ by minimizing a loss function $L(\Theta) = \frac{1}{n}\sum_{t=1}^{n} \|H_t(I_i;\ \Theta) - F_t(\lambda)\|^2$ defined as the average mean squared error between the network prediction and the corresponding ground truth spectrum data, based on the backpropagation algorithm.

In our CNN (**Fig. S5**), our input size is 187x34, with four convolutional layers (20 filters with a size of 3, 40 filters with a size of 4, 64 filters with a size of 3, 128 filters with a size of 3) and pooling layers are stacked. The final output is obtained by flattening the output of the last pooling layer via three dense (fully connected) layers (like the ones found in the MLP). The CNN transforms the input via a sequence of intermediate representations by convolving the input with more learned filter matrices in a convolutional layer and passing the output through a nonlinear pooling operation in a pooling layer.

As shown in **Table S1,** a total of 600 spectra with different peaks and intensities and their corresponding resonance patterns are obtained with experiments. Among these data, 500 spectrums were used as the training data to train the deep network for the proposed method and to calibrate the spectral response function for the conventional method. The remaining 100 spectra were used for testing the trained network and the calibration equations.

The testing spectra obtained from experiment was used as reference. The reconstruction quality was also evaluated quantitatively using normalized mean-squared error (NMSE):

$$NMSE(\hat{x}_i) = \|\hat{x}_i - x_i\|_2^2 / \|x_i\|_2^2 \qquad i=1, 2, \ldots n \tag{4}$$

$\hat{x}_i$ is the reconstruction of the testing patterns using the trained network and the calibration equations, x_i is testing spectra obtained from experiment, and n is the number of testing data. **Fig. S6** shows the mean and standard deviation of the NMSEs of the AI and conventional methods, which are based on a total of 100 testing images. The top of bar is the mean value of each result. Error bar indicates the standard deviation. We also evaluated the reconstruction accuracy using different amount of training data (20%-80%). All these quantitative metrics indicate that it is worthwhile to use AI method for spectrum reconstruction.

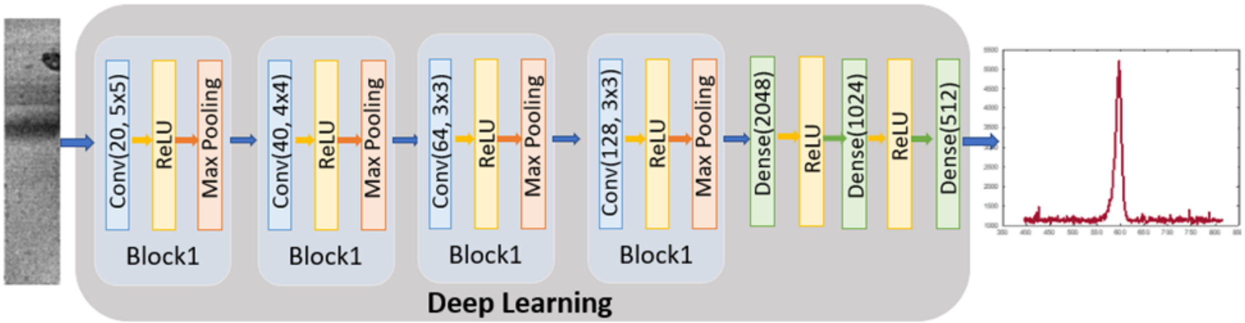

Figure S5. Flowchart of the deep learning spectrum reconstruction method.

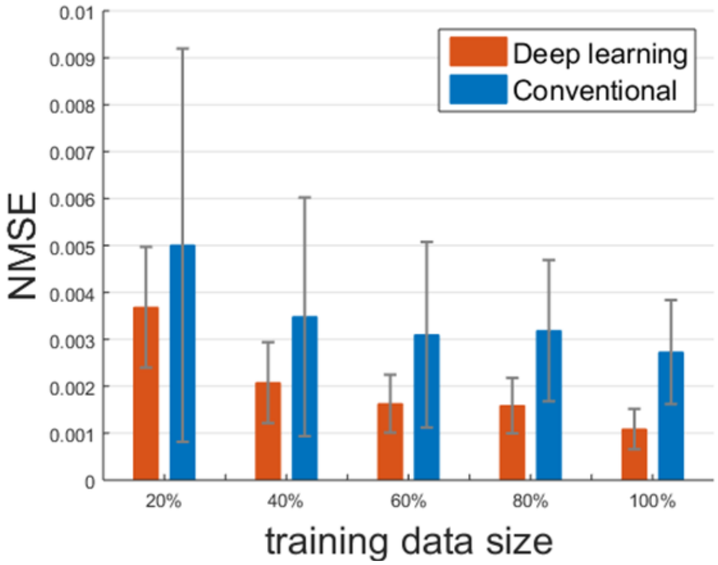

Figure S6. NMSE comparison between the deep learning and conventional reconstruction methods (error bars indicate the standard deviation of measured datasets).

Table S1. Training and testing data parameters for 1D spectrum reconstruction.

<table>
<tr><th>Dataset</th><th>Wavelength (nm)</th><th>Intensity (a.u.)</th><th>Total</th><th>Total</th></tr>
<tr><td>Single wavelength</td><td>435
460
470
490
500
525
595
635
660
770
(10 wavelengths)</td><td>100%
80%
60%
50%
40%
20%
(6 intensities)</td><td>10 × 6 =

60
spectra and images</td><td rowspan="3">60+500+64=

624
spectra and images</td></tr>
<tr><td>Double wavelength</td><td>435 & 460
435 & 470
435 & 490
435 & 500
435 & 525
435 & 595
435 & 635
435 & 660
435 & 770
460 & 525
460 & 595
460 & 635
460 & 660
460 & 770
470 & 525
470 & 595
525 & 635
525 & 660
595 & 635
595 & 660
(20 combinations)</td><td>100% & 100%
100% & 80%
100% & 60%
100% & 40%
100% & 20%
80% & 100%
80% & 80%
80% & 60%
80% & 40%
80% & 20%
60% & 100%
60% & 80%
60% & 60%
60% & 40%
60% & 20%
40% & 100%
40% & 80%
40% & 60%
40% & 40%
40% & 20%
20% & 100%
20% & 80%
20% & 60%
20% & 40%
20% & 20%
(25 intensity pairs)</td><td>20 × 25 =

500
spectra and images</td></tr>
<tr><td>Triple wavelength</td><td>460 & 525 & 635
460 & 525 & 770
470 & 525 & 635
470 & 525 & 770
460 & 595 & 635
460 & 595 & 770
470 & 595 & 635
470 & 595 & 770
(8 combinations)</td><td>100% & 100% & 100%
80% & 100% & 100%
100% & 80% & 100%
100% & 100% & 80%
60% & 100% & 100%
100% & 60% & 100%
100% & 100% & 60%
100% & 60% & 60%</td><td>8 × 8 =

64
spectra and images</td></tr>
</table>

		**(8 intensity pairs)**		

Note S5: Reconstruction of broadband spectra

Reconstruction of arbitrary spectra from sun light and halogen lamp will require sufficient training data including combinations of many different narrow and broadband spectral features. This type of spectral tunability for training data collection is not available in our laboratory at this stage. Here we employed the LED light source to demonstrate a broadband spectrum reconstruction. This LED light source allows for a combination of multiple LEDs to construct more complicated spectra. As a result, the spectral feature is different from those spectra from individual LEDs. As shown in **Table S2**, we collected training data based on different combination data sets and then tested three-peak spectra which were not included in the training data sets.

Table S2. Parameters of the training data collected for broadband spectrum reconstruction.

Dataset	Wavelength (nm)	Images per Setup	Intensity (a.u.)	Total
**Single wavelength**	470<br>490<br>500<br>525<br>550<br>595<br>635<br>660<br>740		100%<br>90%	9 × 50 × 2 =<br>**900 spectra and images**
**Double wavelength**	470 & 525<br>470 & 550<br>470 & 595<br>470 & 635<br>470 & 660<br>470 & 740<br>490 & 525<br>490 & 550<br>490 & 595<br>490 & 635<br>490 & 660<br>490 & 740<br>500 & 525<br>500 & 550<br>500 & 595<br>500 & 635<br>500 & 660<br>500 & 740<br>525 & 635<br>525 & 660<br>525 & 740<br>550 & 635<br>550 & 660<br>550 & 740<br>595 & 635<br>595 & 660<br>595 & 740	**50**	100% & 100%<br>100% & 90%<br>90% & 100%<br>90% & 90%	27 × 50 × 4 =<br>**5400 spectra and images**
**Triple wavelength**	470 & 525 & 635<br>470 & 550 & 660<br>470 & 595 & 740<br>490 & 525 & 635<br>490 & 525 & 660<br>500 & 550 & 660		100% & 100% & 100%<br>100% & 100% & 90%<br>100% & 90% & 100%<br>100% & 90% & 90%<br>90% & 100% & 100%<br>90% & 100% & 90%	7 × 50 × 8 =<br>**2800 spectra and images**

	500 & 595 & 635		90% & 90% & 100%<br>90% & 90% & 90%	

Based on this training dataset, we collected four different sets of three-wavelength combinations with different intensities for testing, which were not included in the training datasets (see parameters in **Table S3**). Fig. 2d in the main text shows four representative reconstructed spectra (red dots). Compared with the measured spectra (solid blue lines), one can see that the spectral features (especially the feature at the overlapped regime) were well predicted.

Table S3. Parameters of the testing data collected for broadband spectrum reconstruction.

Dataset	Wavelength (nm)	Images per Setup	Intensity (a.u.)	Total
**Triple wavelength**	490 & 550 & 635<br>490 & 550 & 660<br>500 & 550 & 635<br>500 & 550 & 660	**50**	100% & 100% & 100%<br>100% & 100% & 90%<br>100% & 90% & 100%<br>100% & 90% & 90%<br>90% & 100% & 100%<br>90% & 100% & 90%<br>90% & 90% & 100%<br>90% & 90% & 90%	4 × 50 × 8 =<br><br>**1600 spectra and images**

The square root of sum-of-squares of the testing spectra obtained from experiment was used as reference. The reconstruction quality was also evaluated quantitatively using normalized mean-squared error (NMSE) (see Note S4). **Fig. S7** shows the mean and standard deviation of the NMSEs of the AI and conventional methods, which are based on a total of 1600 testing images. The top of bar is the mean value of each result. Error bar indicates the standard deviation. As a result, the mean of NMSEs is 0.0026, standard deviation of NMSEs is 0.0013, confirming the accurate reconstruction of broadband spectra with sufficient training.

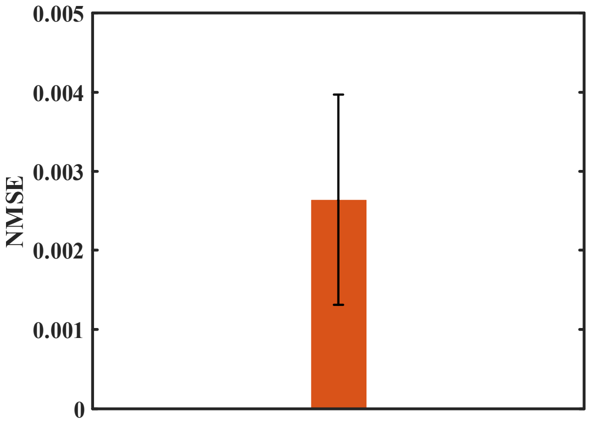

Figure S7. NMSE for the reconstructed broadband spectra using the proposed deep learning method (error bars indicate the standard deviation of measured datasets).

Note S6: Spectral resolution of reconstructed spectra

We captured 10,000 images of the rainbow chip under the illumination of narrowband incidence from 600 nm to 650 nm with a step size of 0.1 nm. To illustrate the minimum peak shift that can be resolved by the smart rainbow spectrometer system, we choose different wavelength shift steps of 0.5 nm, 0.2 nm and 0.1 nm for training and test data. As shown in **Table S4**, after we collected data with different steps, 80% of spectra and images were first used as training data and the rest data for testing. Representative reconstructed spectra are shown in Fig. 2d in the main text. The accuracy of the wavelength peak was 87.0% for the step of 0.5 nm, 81.0% for 0.2 nm and 40.0% for 0.1 nm. When the training data were increased to 90% of the spectra and images, the accuracy was increased to 95.0% for the step of 0.5 nm, 90.0% for 0.2 nm and 69.5% for 0.1 nm. This preliminary analysis demonstrated that the accuracy of the reconstructed spectra can be improved by increasing the training data set.

Table S4. Training and testing data parameters for spectral resolution.

**Step size**	**Range (nm)**	**Images per Setup**	**Intensity (a.u.)**	**Total**	**Training data**	**Testing data**	**Accuracy**
**0.5 nm**	600.0 nm-650.0 nm	**20**	100%	2 × 50× 20 = **2000 spectra and images**	2000×80%= 1600 spectra and images	2000×20%= 400 spectra and images	348/400= 87.0%
					2000×90%= 1800 spectra and images	2000×10%= 200 spectra and images	190/200= 95.0%
**0.2 nm**	600.0 nm-650.0 nm		100%	5 × 50 × 20 = **5000 spectra and images**	5000×80%= 4000 spectra and images	5000×20%= 1000 spectra and images	810/1000= 81.0%
					5000×90%= 4500 spectra and images	5000×10%= 500 spectra and images	450/500= 90.0%
**0.1 nm**	600.0 nm-650.0 nm		100%	10 × 50 × 20 = **10000 spectra and images**	10000×80%= 8000 spectra and images	10000×20%= 2000 spectra and images	800/2000= 40.0%
					10000×90%= 9000 spectra and images	10000×10%= 1000 spectra and images	695/1000= 69.5%

Note S7: Reconstruction of two-peak spectra

Reconstruction of two adjacent peaks will require sufficient training data including single spectra and combinations of many different narrow spectral features. Here we employed the SuperK FIANIUM (NKT Photonics) to demonstrate a two-peak spectrum reconstruction. This light source allows for a combination of multiple narrowband light to construct complicated spectra. As shown in **Table S5**, we collected training data based on single spectra and double combination data sets and then tested spectra that were not included in the training data sets.

We first captured 501 single wavelength images of the rainbow chip from 596.8 nm to 646.8 nm with a step size of 0.1 nm. To illustrate the two narrow peaks that can be resolved by the smart rainbow spectrometer system, we fixed one peak at 596.8 nm and sweep the second wavelength from 596.8 nm to 646.8 nm with a step size of 0.1 nm. As shown in **Table S5**, all single wavelength images and 80% of double images and spectra were used as the training data. The rest 20% double-peak images and spectra were used for testing. The accuracy was defined if the peaks of the reconstructed spectra and the measured ones have the same peak position.

Table S5. Training and testing data parameters for two-peak spectrum reconstruction.

<table>
<tr><th>Dataset</th><th>Wavelength (nm)</th><th>Step size</th><th>Total</th><th>Training data</th><th>Testing data</th><th>Accuracy</th></tr>
<tr><td>Single wavelength</td><td>596.8 nm
596.9 nm
597.0 nm
.
.
.
646.7 nm
646.8 nm</td><td rowspan="2">0.1 nm</td><td>501 spectra and images</td><td rowspan="2">Single
+
80% Double

501
+
501×80%
= 901
spectra and images</td><td rowspan="2">20% Double

501×20%
= 100
spectra and images</td><td rowspan="2">94/100
=
94%</td></tr>
<tr><td>Double wavelength</td><td>596.8 nm & 596.9 nm
596.8 nm & 597.0 nm
.
.
.
596.8 nm & 646.7nm
596.8 nm & 646.8nm</td><td>501 spectra and images</td></tr>
</table>

Note S8: Resolving adjacent two peaks using Rayleigh criterion

According to Rayleigh criterion widely used for optical resolution in imaging applications, a dip with the intensity of approximately 81% of the peak intensity is frequently used as the baseline. As shown in **Fig. S8**, no obvious dip can be observed in DL-reconstructed line for the spectra of 596.8+598.4 nm and 596.8+598.6 nm. When the second peak was tuned to 598.8 nm, the intensity line profile of the DL-reconstructed spectrum shows a valley-to-peak intensity of 79.1%, meeting the Rayleigh criterion. As a result, the resolution of the proposed intelligent spectrometer in resolving adjacent peaks is at least 2 nm.

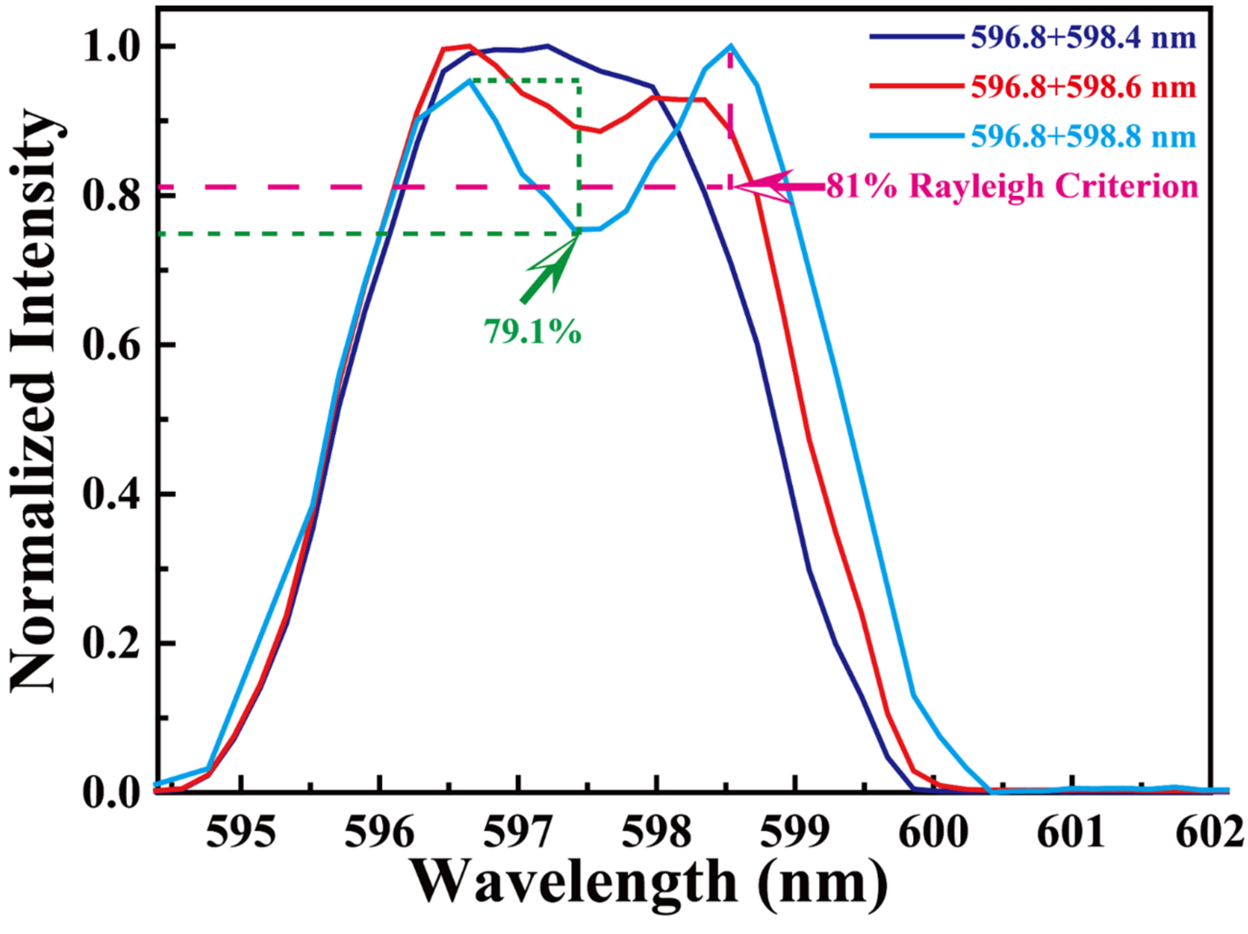

Figure S8. Reconstruction of two adjacent peaks to determine the resolution using the Rayleigh criterion. DL-reconstructed spectra are for 596.8+598.4 nm, 596.8+598.6 nm, and 596.8+598.8 nm, respectively. The peaks of 596.8+598.8 nm with a drop to 79.1% intensity (see the green arrow) satisfy the Rayleigh criterion (i.e., 81%).

Note S9: Fabrication of the 2D rainbow chip

Fabrication of the 2D rainbow chip followed the same procedure as the 1D rainbow chip (i.e., deposition of a 300 nm Ag film on glass substrate via EB deposition, followed by FIB milling of the grating patterns). The pattern for the 2D chip was designed on the same NPVE software as the 1D chip pattern. The period of the 2D grating pattern varied from 439 nm to 739 nm in both directions. Formation of the gradient followed the same method used for the 1D chip fabrication (i.e., assembly of six-groove groups, with the period increasing by 10 nm along the length of the pattern). The final groove pattern was applied horizontally (left to right) and then overlapped vertically (top to bottom), from shortest period to longest period in both directions, to form the 2D pattern. The area of the entire 2D grating was 7735.2 μm^2, with the length of each graded pattern being 87.95 μm and the height of the grooves matched to create a square shaped structure. Refer to Fig. 4a-c in the main text to see SEM images of the 2D graded grating structure.

Note S10: Modeling of the 2D rainbow chip

The electric field distribution on the 2D graded grating are calculated using the commercial software Lumerical FDTD Solutions. Perfectly matched layers are applied at boundaries to absorb the outgoing waves. As shown in **Fig. S9a**, a plane wave with the polarization angle of 45° was introduced on the 2D chirped grating (with the width of 200 nm and depth of 35 nm). The period of the grating changes from 440 nm to 740 nm along two directions with the step size of 10 nm. Under the illumination at the narrow band incident wavelength 595 nm, 636 nm, and 660 nm, the electric-field distributions in the near-field are recorded (see **Fig. S9b-d**). The cross bar with two arms can be clearly seen in the near field. As the wavelength increases, one can see that the crossbar pattern shifts towards the larger period region, agreeing well with the experiment observation in Fig. 4d.

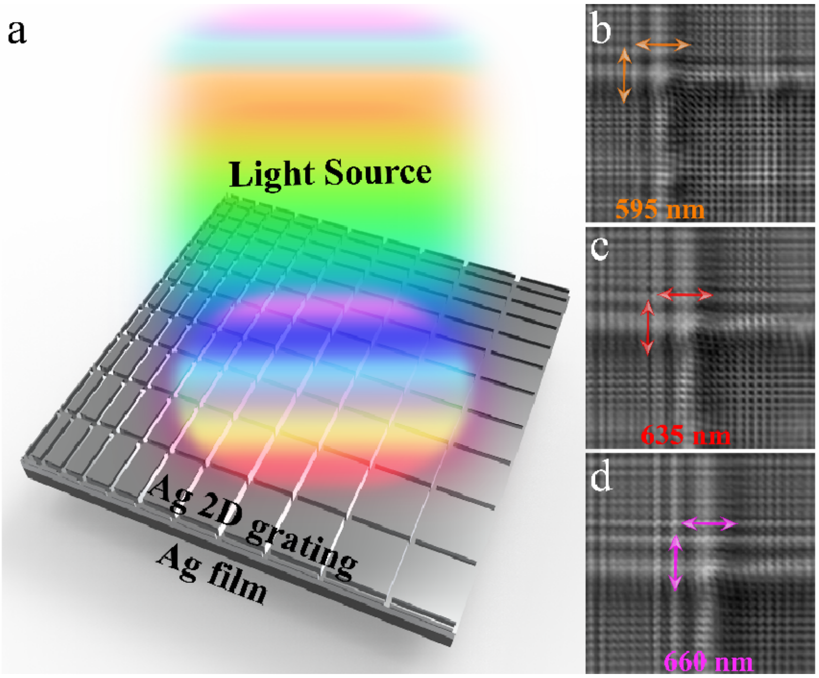

Figure S9. Numerical modeling of the 2D chirped grating. (a) Schematic of graded surface grating structure illuminated by an incident plane wave. (b)-(d) The simulated top-view electric-field distributions in near field (i.e., 3.5 µm above the grating surface) at the incident wavelengths of (b) 595 nm, (c) 635 nm, and (d) 660 nm, respectively.

Note S11: Spectral range of a given 2D rainbow chip

The spectral range of the graded grating is tunable by the geometric parameters of the surface grating. According to our earlier investigation, the spectral range was tuned from visible [26], to infrared [25] and even THz domain [24]. For a given grating structure (e.g., the grating used in Fig. 4 with the period ranging from 439 nm to 739 nm), the spectral range can be directly characterized. As shown in **Fig. S10**, we tuned the incident wavelength and revealed that its spectral range is from about 470 nm to 770 nm in wavelength.

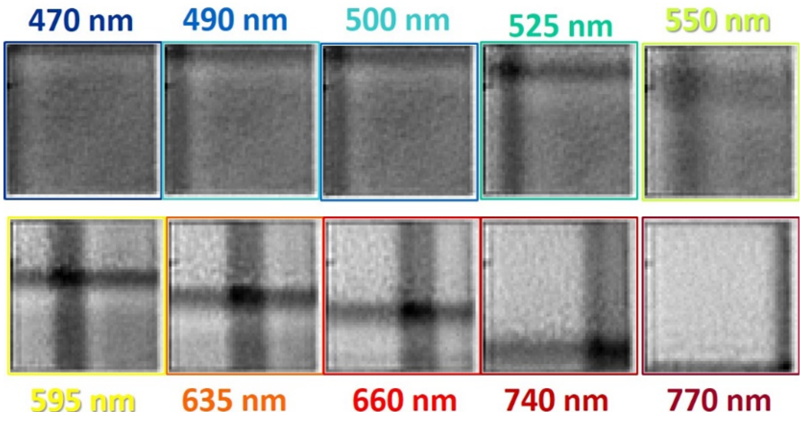

Figure S10. Spectral range determination of the 2D graded grating. The period is tuned from 439 nm to 739 nm.

Note S12: Deep learning method for polarized spectra reconstruction

For polarization-dependent spectrum reconstruction, we use two networks to consider the 2D geometric parameters of the graded grating. As shown in **Fig. S11**, the outputs of networks indicated the vertical and horizontal information in the corresponding pattern. The input size is 190×190, and all the details of the layers are the same as those in **Fig. S5**.

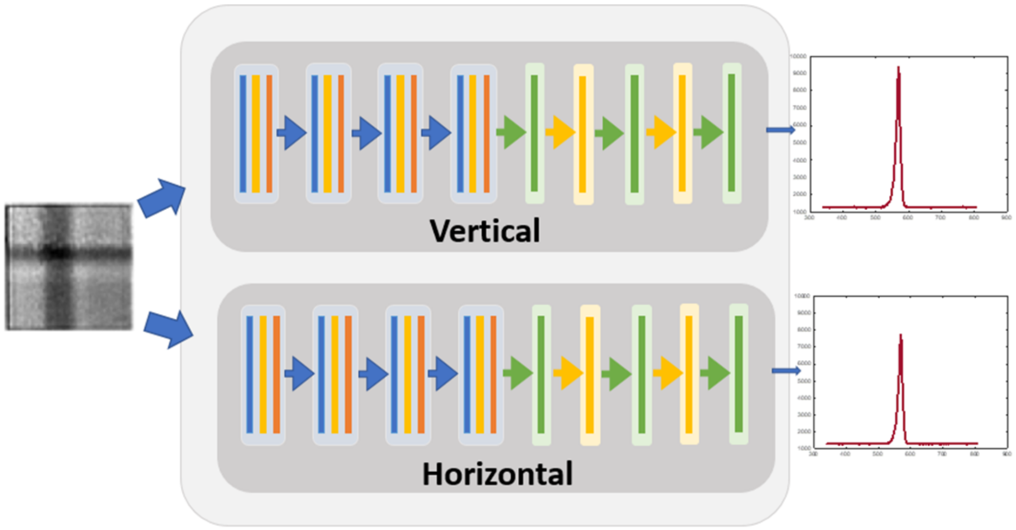

Figure S11. Flowchart of the polarized spectrum reconstruction.

Note S13: Training and testing procedure for spectrum reconstruction of the image-based spectrometer

Training and testing data for spectrum reconstruction consisted of images of the rainbow chip, and the corresponding spectrum measured by a conventional visible spectrometer, for all illumination conditions. These data were broken down into three sets labeled as Single, Double, and Triple wavelength based on the illumination conditions used to categorize them (see **Table S6**). Single wavelength data was collected using 490 nm, 595 nm, 635 nm, and 660 nm illumination. For each setup, illumination was set to 100%, 80%, and 60% of the maximum intensity available for that wavelength by the LED light source, respectively. For the Double wavelength dataset, five wavelength combinations were used for illumination. Each combination was a pair of two wavelengths, picked from the pool of wavelengths used in the Single wavelength dataset. Each wavelength pair was illuminated using combinations of 80%-80%, 80%-60%, 60%-80%, and 60%-60% of the maximum intensity available for the chosen wavelengths. Triple wavelength followed a similar approach, this time with two combinations of three-wavelength pairs. These triple-wavelength pairs were illuminated under combinations of 100%-100%-100%, 80%-100%-100%, 100%-80%-100%, and 100%-100%-80% maximum intensity available for each wavelength. A set of 15 polarization states ranging from 0° to 90° was applied to all datasets. These 15 polarization states were characterized by $= \frac{90}{14} * N$, where θ is the polarization angle and $N \sim [0, 14]$ is an index used for calculation. As a result, a total of 180, 300, and 120 spectra-image pairs were collected for the Single, Double, and Triple wavelength datasets, respectively. This gives a grand total of 600 measured spectra and corresponding images. Training data consisted of all Single and Double wavelength conditions, as well as 20 conditions for the 490-595-660 nm Triple wavelength pair. Thus, testing data consisted of the remaining 40 conditions for the 490-595-660 nm wavelength pair, and all conditions for the 490-595-635 nm wavelength pair.

Table S6. Parameters of the training and testing data collected for spectrum reconstruction.

Dataset	Wavelength (nm)	Polarization (deg.)	Intensity (a.u.)	Total

<table>
<tr><td>Single wavelength</td><td>490
595
635
660
(4 wavelengths)</td><td rowspan="3">0
6.43
12.86
19.29
25.71
32.14
38.57
45
51.43
57.86
64.29
70.71
77.14
83.57
90
(15 polarizations)</td><td>100%
80%
60%
(3 intensities)</td><td>4 × 15 × 3 =
180
spectra and images</td></tr>
<tr><td>Double wavelength</td><td>490 & 595
490 & 635
595 & 635
490 & 660
595 & 660
(5 combinations)</td><td>80% & 80%
80% & 60%
60% & 80%
60% & 60%
(4 intensity pairs)</td><td>5 × 15 × 4 =
300
spectra and images</td></tr>
<tr><td>Triple wavelength</td><td>490 & 595 & 635
490 & 595 & 660
(2 combinations)</td><td>100% & 100% & 100%
80% & 100% & 100%
100% & 80% & 100%
100% & 100% & 80%
(4 intensity pairs)</td><td>2 × 15 × 4 =
120
spectra and images</td></tr>
</table>

Note S14: Deep learning method for polarization state prediction

ML-based classification [R6] is an important functionality for polarization state prediction. For a single wavelength, we consider a combination of convolutional layers and fully connected layers to design a specific classifier, as shown in **Fig. S12.** The input is an SEM image of a preliminary sample, as described before. A total of four convolutional layers with ReLU and pooling layers were used in our network. The final output from the fourth convolution block flattened to a low dimensional data representation by three dense layers. Different from reconstruction, we added dropout layers after the dense layers due to the size of the output layer being much smaller than for reconstruction. Eventually, the third dense layer is used to calculate the probability of each label and assign the most likely one to the given input. In this study, a network that can classify multiple wavelengths has been built as shown in **Fig. S13** This network includes nine single wavelength classifiers whose spectral range cover from 470 nm to 740 nm. A total of 31,900 Air and deionized (DI) water images with 29 different polarizations (45°±14°) are obtained for training data. The trained DL model was then tested using 660 images of the chip under similar illumination conditions. The input size is 190×190 and a total of 30 labels are used as the output. The first label of 30 has been used to decide if the input image had the information of the specific wavelength. If the first label of output is 1, it means there is no information about the corresponding wavelength. The rest 29 of the 30 have been labeled as the 29 different polarizations. The input of **Fig. S13** is 470 nm-595 nm-740 nm set at 45 degrees with 30% glucose. The first label of other wavelengths has a high probability due to there being no information except for 470 nm, 595 nm, and 740 nm. The prediction state of the three wavelengths is 29, 23 and 20, which satisfies Fig. 5g.

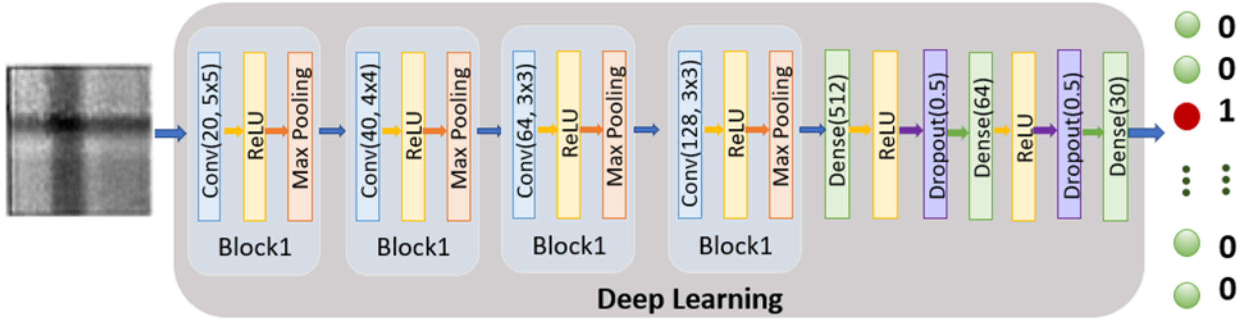

Figure S12. Flowchart of the single wavelength polarization prediction.

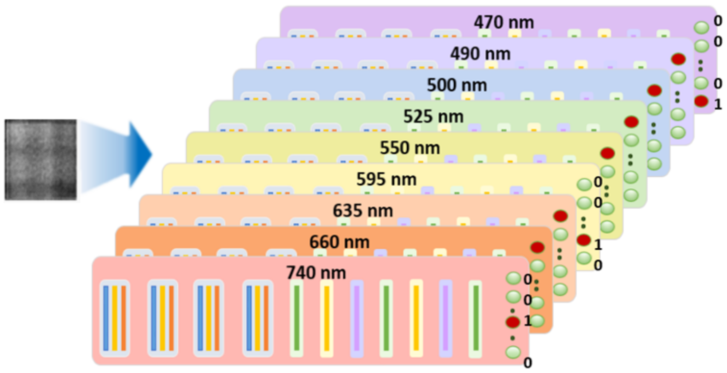

Figure S13. Flowchart of the multiple wavelength polarization predictions.

Note S15: Training and testing procedure for ORD sensing of the image-based spectrometer

To begin modeling the DL algorithm for ORD sensing, training and testing data under single-wavelength illumination was collected. Illumination peaks at 470, 490, 500, 525, 550, 595, 635, 660, and 740 nm were used. These nine peaks were selected and controlled using the digital controller of the cool LED light source used in this study.

For the training data, air and DI water were used as the samples to initiate all light-matter interactions. A Thorlabs polarizer (PRM1) was used to control the polarization direction of incident light. The polarizer has a rotational scale engraved along its wheel for accurate measurement of the polarization direction, with a micrometer attachment for fine-tune adjustment. Due to the high precision measurements offered by these features, we recorded the training data with the incident polarizer direction tuned from 31° to 59° by 29 steps with a step size of 1°. For every combination of these parameters, 50 images were taken to reduce the effect of noise. This resulted in a dataset of 9 × 29 × 2 × 50 = 26,100 training images.

For testing data, aqueous glucose solutions of 2%, 10%, and 30% concentration were used. Instead of a range of polarization states, all testing data was collected at a single polarization state of 45°. This is because air and water do not introduce optical rotation, while glucose solutions will introduce an optical rotation depending on their concentration. Since the training data consists of images taken using air and DI water, the DL algorithm can only make predictions of the polarization state under the assumption that either of these samples are used. At 45°, the intensity of both arms in the crossbar pattern are equal, and changes in

polarization are most noticeable. By collecting testing data at this single polarization state, predictions of the polarization made by the DL algorithm can be directly translated into measurements of the optical rotation induced by the sample. For testing data, 20 images were taken for each combination of parameters. This resulted in a total of 9 × 1 × 3 × 20 = 540 images of testing data. See **Table S7** for an overview of the parameters used for the single-wavelength illumination.

Table S7. Training and testing data parameters for single-peak illumination for ORD sensing.

	**Wavelength**	**Polarization States**	**Samples/ Solutions**	**Images per Setup**	**Total Images**
**Training Data**	470 nm<br>490 nm<br>500 nm<br>525 nm<br>550 nm<br>595 nm<br>635 nm<br>660 nm<br>740 nm<br>**(9 wavelengths)**	45° ± 14°<br>**(29 polarizations)**	Air, DI Water<br>**(2 samples)**	**50**	9 × 29 × 2 × 50 =<br>**26,100**
**Testing Data**		45°<br>**(1 polarization)**	2%, 10%, 30% glucose<br>**(3 solutions)**	**20**	9 × 1 × 3 × 20 =<br>**540**

To achieve multi-peak spectral analysis and ORD sensing with the proposed compact rainbow spectrometer, additional sets of training and testing data was collected under double-wavelength and triple-wavelength illumination conditions. An overview of the parameters used for collecting the double-wavelength and triple-wavelength data are listed in **Table S8** and **Table S9**, respectively.

For the dataset of the double-wavelength illumination, a pair of 525 nm and 660 nm illumination was used. Training data was collected using air and DI water samples over a polarization range of 45° ± 14°. For each setup, 50 images of the on-chip resonance pattern were taken. Testing data was collected using 2%, 10%, and 30% aqueous glucose solutions at only 45° polarization, with 20 images taken for each setup. This gave a total of 1 × 29 × 2 × 50 = 2,900 training images and 1 × 1 × 3 × 20 = 60 testing images.

For the dataset of the triple-wavelength illumination, a pair of 470 nm, 595 nm, and 740 nm illumination was used. Training and testing data parameters followed the same format as for the dataset of the double-wavelength illumination, resulting in the same amount of training and testing images.

Table S8. Training and testing data parameters for double-peak illumination for ORD sensing.

	**Wavelength**	**Polarization States**	**Samples/ Solutions**	**Images per Setup**	**Total Images**
**Training Data**	525 nm<br>&<br>660 nm<br>**(1 pair)**	45° ± 14°<br>**(29 polarizations)**	Air, DI Water<br>**(2 samples)**	**50**	1 × 29 × 2 × 50 =<br>**2,900**

**Testing Data**	525 nm<br>&<br>660 nm<br>**(1 pair)**	45°<br>**(1 polarization)**	2%, 10%, 30% glucose<br>**(3 solutions)**	**20**	1 × 1 × 3 × 20 =<br><br>**60**

Table S9. Training and testing data parameters for triple-peak illumination for ORD sensing.

	**Wavelength (nm)**	**Polarization States**	**Samples/ Solutions**	**Images per Setup**	**Total Images**
**Training Data**	470 nm<br>&<br>595 nm<br>&<br>740 nm<br>**(1 pair)**	45° ± 14°<br>**(29 polarizations)**	Air, DI Water<br>**(2 samples)**	**50**	1 × 29 × 2 × 50 =<br><br>**2,900**
**Testing Data**	470 nm<br>&<br>595 nm<br>&<br>740 nm<br>**(1 pair)**	45°<br>**(1 polarization)**	2%, 10%, 30% glucose<br>**(3 solutions)**	**20**	1 × 1 × 3 ×20 =<br><br>**60**

Table S10. Deviation of reconstructed angles at different wavelengths in Fig. 5f-g in the main text.

Double-peak deviation:

Glucose concentration	525 nm	660 nm
2%	0.25°	0.08°
10%	0.27°	0.28°
30%	0.26°	0.07°

Triple-peak deviation:

Glucose concentration	490 nm	595 nm	740 nm
2%	0.20°	0.19°	0.10°
10%	0.45°	0.32°	0.20°
30%	0.09°	0.34°	0.08°

Note S16: Experimental measurement of the ORD using conventional methods

Experimental measurement of the ORD induced by the 2%, 10%, and 30% glucose solutions was performed using conventional methods. Our setup was based on the design in Fig. 5b of the main text. The cool LED was used to set light incident onto a polarizer. This first polarizer was fixed at 45° to control the polarization of the input illumination. Glucose solutions of 2%, 10%, and 30% concentrations were placed as samples after the first polarizer for the incident light to interact with. After leaving the sample, the output illumination passed through a second polarizer, called an analyzer, and finally a benchtop spectrometer used as a detector. The output polarization was determined by adjusting the polarization of the analyzer until the intensity of the light measured by the spectrometer was maximized. These measurements were then compared to the fixed initial polarization to calculate the optical rotation. Spectral peaks at 470 nm, 490 nm, 500 nm, 525 nm, 550 nm, 595 nm, 635 nm, 660 nm, and 740 nm were used to capture the full ORD for each solution. Fitted curves of the data were then constructed and plotted for direct comparison with the predictions made by the DL algorithm.

Note S17: Comparative figures-of-merit (FOMs) of the proposed scheme with previous works

To highlight the advantages and limitations of the proposed 'rainbow' chip spectrometer, we created a table listing common figures-of-merit (FOMs) of our proposed scheme in comparison with those from several references. Please refer to **Table S11** below to see these comparisons.

Table S11. Comparative figures-of-merit (FOMs) of the scheme proposed in this paper and schemes from several references. *CV stands for Coefficient of Variance, which is defined by the referenced article as the ratio of the standard deviation to the mean of the photocurrent measurements. **DOP is defined by the reference as Degree of Polarization. ***Errors are listed as the average peak localization error, bandwidth error, height error, and MSE of the reconstructions, respectively. ****Data for this block was not included in the reference.

Reference	Design	Fabrication	Device Size	Resolution	Operational Range	Error	Functionality
This work	2D plasmonic chirped grating on metal film	Metal deposition, focus ion-beam milling	87.95 × 87.95 μm^2	2 nm	470 – 770 nm	0.0026 ± 0.0013 NMSE	Spectral reconstruction,

							**polarization prediction**
[2]	195 colloidal quantum dot filters coupled to a camera	CQD growth, printing of CQD/PVD solution, integration onto CCD	8.5 × 6.8 mm	2 nm	390 – 690 nm	0.022 std. dev.	Spectral reconstruction
[3]	Single compositionally engineered nanowire	Annealing, e-beam lithography, plasma treatment, atomic layer deposition, metal deposition and liftoff	0.5 × 75 µm	10 nm	500 – 630 nm	2% CV*	Spectral imaging, spectral reconstruction
[4]	Photonic crystal slabs on CMOS sensors	E-beam lithography, reactive ion etching	210 × 210 µm	≈ 1 nm	550 – 750 nm	< 0.05 MSE	Hyperspectral imaging, spectral reconstruction
[8]	Photonic crystal structure coupled with photodetectors	Deep UV lithography, plasma etching	1 × 0.3 mm	-****	-90° – +90°	0.07 DOP std. deviation**	Polarization analysis
[9]	Compressive spectroscopy via thin film filter array	E-beam deposition, film deposition, photolithography	2.5 × 2.5 mm	-****	500 – 1000 nm	0.016 MSE	Spectral reconstruction
[15]	Metasurfaces composed of freeform shaped meta-atoms	E-beam lithography, ICP etching, wet etching, PDMS transfer	8 × 6.4 mm	0.5 nm	450 – 750 nm	0.024 nm std. dev.	Spectral reconstruction, ultraspectral imaging
[16]	Plasmonic spectral encoder chip comprised of 252 nanohole arrays	E-beam lithography, imprint molding, e-beam evaporation	4.8 × 3.6 mm	-****	480 – 750 nm	0.19 nm<br>0.18 nm<br>7.60%<br>7.77×10^{-5}<br>***	Spectral reconstruction

Supplementary References

[R1] Yang, Z. et al. Single-nanowire spectrometers. Science **365**, 1017-1020 (2019).

[R2] Bao, J. & Bawendi, M. G. A colloidal quantum dot spectrometer. Nature **523**, 67-70 (2015).

[R3] Wang, Z. et al. Single-shot on-chip spectral sensors based on photonic crystal slabs. Nature Communications **10**, 1020-1020 (2019).

[R4] Liang, D., Cheng, J., Ke, Z. & Ying, L. Deep Magnetic Resonance Image Reconstruction: Inverse Problems Meet Neural Networks. IEEE Signal Processing Magazine 37, 141-151 (2020).

[R5] Wang, S. et al. Accelerating magnetic resonance imaging via deep learning. IEEE 13th International Symposium on Biomedical Imaging (ISBI). 514-517 (2016).

[R6] K. M. Sindhoora et al. Machine-learning-based classification of Stokes-Mueller polarization images for tissue characterization. J. Phys.: Conf. Ser. 1859 012045 (2021).
